# Glucose Levels in At-risk Newborns (GLEAN): a prospective cohort study on glucose profiles in infants at risk of hypoglycemia

**DOI:** 10.3389/fendo.2025.1599366

**Published:** 2025-08-08

**Authors:** Fabian Yap, Daniel Chan, Ruther Teo Zheng, Lakshmi Shandra Bos, Victor Samuel Rajadurai, Suresh Chandran

**Affiliations:** ^1^ Endocrinology Service, KK Women’s and Children’s Hospital, Singapore, Singapore; ^2^ Duke-NUS Medical School, National University of Singapore, Singapore, Singapore; ^3^ Lee Kong Chian School of Medicine, Nanyang Technological University, Singapore, Singapore; ^4^ Department of Neonatology, KK Women’s and Children’s Hospital, Singapore, Singapore

**Keywords:** neonatal endocrinology, hypoglycemia, glucose, neonatology, endocrinology

## Abstract

**Objective:**

To describe glucose patterns in at-risk infants, determine the incidence of hypoglycemia across different risk groups, and evaluate the impact of combined risk factors on odds of developing hypoglycemia.

**Study design:**

This prospective cohort study was conducted at KK Women’s and Children’s Hospital from 16 December 2019 to 16 March 2020, during which 2,564 babies were born. Of these, 701 were identified as at-risk of hypoglycemia based on predefined clinical criteria: infants of diabetic mothers (IDM), term infants with birth weight >4000 g or <2600 g, preterm infants, and infants of obese mothers (IOM). Risk group classification was refined using INTERGROWTH-21^st^ standards, and infants were further stratified by the presence of single or combined risk factors. Complete glucose measurements at 2, 6, 12, 18, and 24 hours were available for 670 infants (95.6%). The primary outcomes were glucose trends and the incidence of hypoglycemia, defined as blood glucose < 3.0 mmol/L.

**Results:**

Mean glucose levels stabilized between 3.8 and 4.0 mmol/L by 24 hours. The highest incidence of hypoglycemia was observed in single risk factor SGA infants (22.6%), followed by IOM (16%), a group less studied in hypoglycemia risk assessments. This was comparable to the incidence seen in IDM (13.0%). In contrast, single risk factor LGA infants exhibited the lowest incidence of hypoglycemia (6.2%). Infants with combined risk factors had a higher incidence of hypoglycemia compared to those with a single risk factor (18.5% vs 15.9%) and showed higher odds of hypoglycemia compared to those with a single risk factor (OR 2.47; 95% CI: 0.98–6.08, p = 0.049).

**Conclusions:**

Glucose trajectories and hypoglycemia risks differ across clinically defined at-risk groups, underscoring the importance of targeted screening and management protocols. Stratifying infants by single and combined risk factors provided additional insight that may support future refinements to neonatal hypoglycemia clinical guidelines.

## Introduction

Neonatal hypoglycemia poses a critical challenge in newborn care, particularly for infants already navigating the complexities of being born small-for-gestational-age (SGA), large-for-gestational-age (LGA), preterm (PT), or infants of mothers with diabetes (IDM). These vulnerable infants face greater risks due to their limited glycogen stores, altered insulin regulation, and/or other metabolic factors, which impair their ability to support stable glucose levels. Prolonged or recurrent hypoglycemia in these infants has been associated with long-term neurodevelopmental impairments, including deficits in visual-motor skills and cognitive dysfunction (1–4). The neurodevelopmental risks associated with neonatal hypoglycemia emphasize the need for timely detection and intervention. However, clinical guidelines lack consistency in defining appropriate glucose thresholds for intervention, complicating the management of at-risk infants across various healthcare settings (5–7). While transient dips in blood glucose (BG) are a natural part of metabolic adaptation for healthy term infants (8), those at risk, especially SGA infants, require heightened vigilance and timely intervention. Altogether, at-risk infants have more complex glucose regulation and require more nuanced monitoring and treatment (3, 8–10).

Despite the well-established link between prolonged neonatal hypoglycemia and adverse neurodevelopmental outcomes, there is still no clear consensus on the glucose thresholds that warrant treatment. Clinical guidelines for screening and treatment thresholds vary significantly, leading to inconsistencies in neonatal care (7). For instance, different thresholds for glucose intervention have been proposed by the Pediatric Endocrine Society and the American Academy of Pediatrics, complicating decision-making in clinical practice across various institutions (1, 5). The GLOW Babies study provided critical insights into the glycemic patterns in healthy newborns, showing that glucose levels typically rise to adult levels by 72 hours postpartum, with substantial variability occurring within the first 48 hours of life (11). However, these findings cannot be directly extrapolated to at-risk infants, who may have impaired ketogenesis and reduced ability to utilize alternative energy sources during hypoglycemic episodes (6, 12).

With global obesity rates rising steadily alongside the prevalence of non-communicable diseases, maternal obesity has emerged as a significant yet underexplored factor in neonatal glucose regulation (9, 13, 14). Both pre-gestational and gestational obesity are strongly associated with altered neonatal glucose regulation, even in the absence of maternal diabetes (9). This risk is compounded when maternal obesity coexists with other metabolic conditions, such as insulin resistance and gestational diabetes. Understanding the relationship between maternal obesity and neonatal hypoglycemia is critical, particularly given its interplay with other risk factors like SGA, LGA, or preterm birth (10, 15).

This study aims to address these knowledge gaps by (i) assessing glucose patterns in infants with single versus combined risk factors, (ii) determining the incidence of hypoglycemia across different risk groups, and (iii) evaluating the impact of combined risk factors on the likelihood of hypoglycemia. Understanding glucose regulation in at-risk infants is critical for optimizing the management of neonatal hypoglycemia. By identifying specific glucose patterns and associated risk factors, this study seeks to inform more tailored approaches to glucose surveillance in clinical practice.

## Methods

### Study design and setting

We conducted a prospective cohort study from 16 December 2019 to 16 March 2020 at KK Women’s and Children’s Hospital, Singapore, which delivers approximately 11,000 live births annually. The study was conducted in accordance with the Declaration of Helsinki and was granted exemption from review and informed consent by the SingHealth Institutional Review Board (CIRB 2021/2166) due to its use of de-identified, routinely collected clinical data.

### Eligibility criteria

Inclusion Criteria: Infants were eligible if they met at least one of the following predefined clinical risk criteria for neonatal hypoglycemia, based on institutional protocols (16, 17): (i) infant of a diabetic mother (IDM), including those with pre-existing or gestational diabetes, (ii) term infant with birth weight >4000g, (iii) term infant with birth weight <2600g, (iv) preterm infant, defined as gestational age < 37 weeks, and (v) infant of a mother with obesity (IOM), defined as maternal body mass index ≥ 33 kg/m² (17, 18).

Exclusion Criteria: Infants were excluded if they: (i) had incomplete risk factor data precluding accurate classification, (ii) did not meet the pathway inclusion criteria, (iii) were admitted directly to level 2 and 3 neonatal care for non-hypoglycemia-related reasons.

### Study population and clinical management

All live births during the study period were reviewed for eligibility. Eligible infants identified were enrolled in a clinical hypoglycemia pathway that prioritized skin-to-skin care, early breastfeeding, or administration of 60ml/kg/day of milk feeds, and standardized glucose monitoring. The first BG level was measured at 2 hours of life, followed by subsequent measurements at 6, 12, 18, and 24 hours. This feed-centric approach (16, 17) tailors hypoglycemia management in at-risk infants based on age and BG thresholds. In the first 4 hours, asymptomatic infants are fed within 1 hour and BG checked at 2 hours. An infant with BG ≥ 3.0mmol/l continues with routine feeding; 1.5 - 2.9mmol/L triggers buccal glucose followed by feeds and recheck in 15 minutes; <1.5mmol/L or symptomatic infants receive immediate IV glucose. After 4 hours, thresholds are adjusted: an infant with BG ≥ 3.0mmol/L continues routine care; 2.0 – 2.9 mmol/L prompts buccal glucose and recheck, and < 2.0mmol/L leads to IV glucose. This approach prioritizes feeding while ensuring appropriate and timely interventions for hypoglycemia. Intravenous glucose was administered to symptomatic infants, as well as those with persistent hypoglycemia despite buccal glucose treatment, in accordance with institutional protocol. Early glucose testing (before 2 hours of life) was avoided to prevent unnecessary intervention during the expected glycemic nadir (19–21).

### Data collection

Maternal variables collected included age, ethnicity, parity, first trimester BMI, and pre-existing diabetes or gestational diabetes status. Infant data included gestational age, sex, and birth weight.

Feeding mode during the first 24 hours were documented for all infants, categorized as exclusive breastfeeding, mixed feeding (breast milk and formula), and exclusive formula feeding.

Postnatal birth weight percentiles were calculated using the INTEGROWTH-21st growth standards (IG21) (22). Based on sex, birth weight, and gestational age, infants were reclassified as small-for-gestational-age (SGA: <10th percentile), appropriate-for-gestational-age (AGA: 10th-90th percentile), and large-for-gestational-age (LGA: >90th percentile). AGA infants without other risk factors who were enrolled based on clinical discretion, were classified as “AGA infants not at risk” for analysis.

### Hypoglycemia assessment

Hypoglycemia was defined based on established criteria from (i) the Pediatric Endocrine Society: BG <3.0 mmol/L, and (ii) the American Academy of Pediatrics: BG <2.2 mmol/L before 4 hours of life, or <2.6 mmol/L between 4–24 hours of life (1, 5).

In our study cohort, hypoglycemia incidence was assessed at predefined thresholds (<3.0 mmol/L, <2.6 mmol/L, and <2.2 mmol/L) across multiple prefeed time points (2, 6, 12, 18, and 24 hours post-birth) to capture both early and late glycemic patterns. These thresholds were selected not only to determine the incidence of hypoglycemia but also to stratify its severity, offering a more nuanced understanding of glycemic trends across different at-risk subgroups.

Blood glucose levels were measured using point-of-care glucose meters (FreeStyle Optium Neo, Abbott Diabetes Care Inc., USA). If a BG level <3.0 mmol/L was detected, a venous sample was obtained via venepuncture and measured using the same point-of-care glucose meter, with the venous value recorded. The glucose meters were maintained monthly throughout the study, and each meter underwent control solution testing with each new box of test strips to ensure consistent performance and quality control.

### Statistical analysis

Statistical analyses were performed using R programming. Maternal and infant characteristics were summarized as frequencies (percentages) for categorical variables and as means (standard deviations) for continuous variables. Differences between groups were analyzed using χ² or Fisher’s exact tests for categorical data, and independent t-tests or one-way analysis of variance (ANOVA) for continuous variables.

Glucose trends over time were compared across feeding groups (breastfeeding, mixed feeding, and formula feeding) to assess potential feeding-related effects. Results are reported as odds ratios (OR) with 95% confidence intervals (CI). We examined the Q-Q plots and the skewness of the histogram for each variable to determine if the variable has a normal distribution. We conducted the Bartlett’s test to assess the equality of variance between groups for each variable. All tests were two-sided, with statistical significance set at p < 0.05.

## Results

### Study cohort and participant characteristics

**Figure 1:**
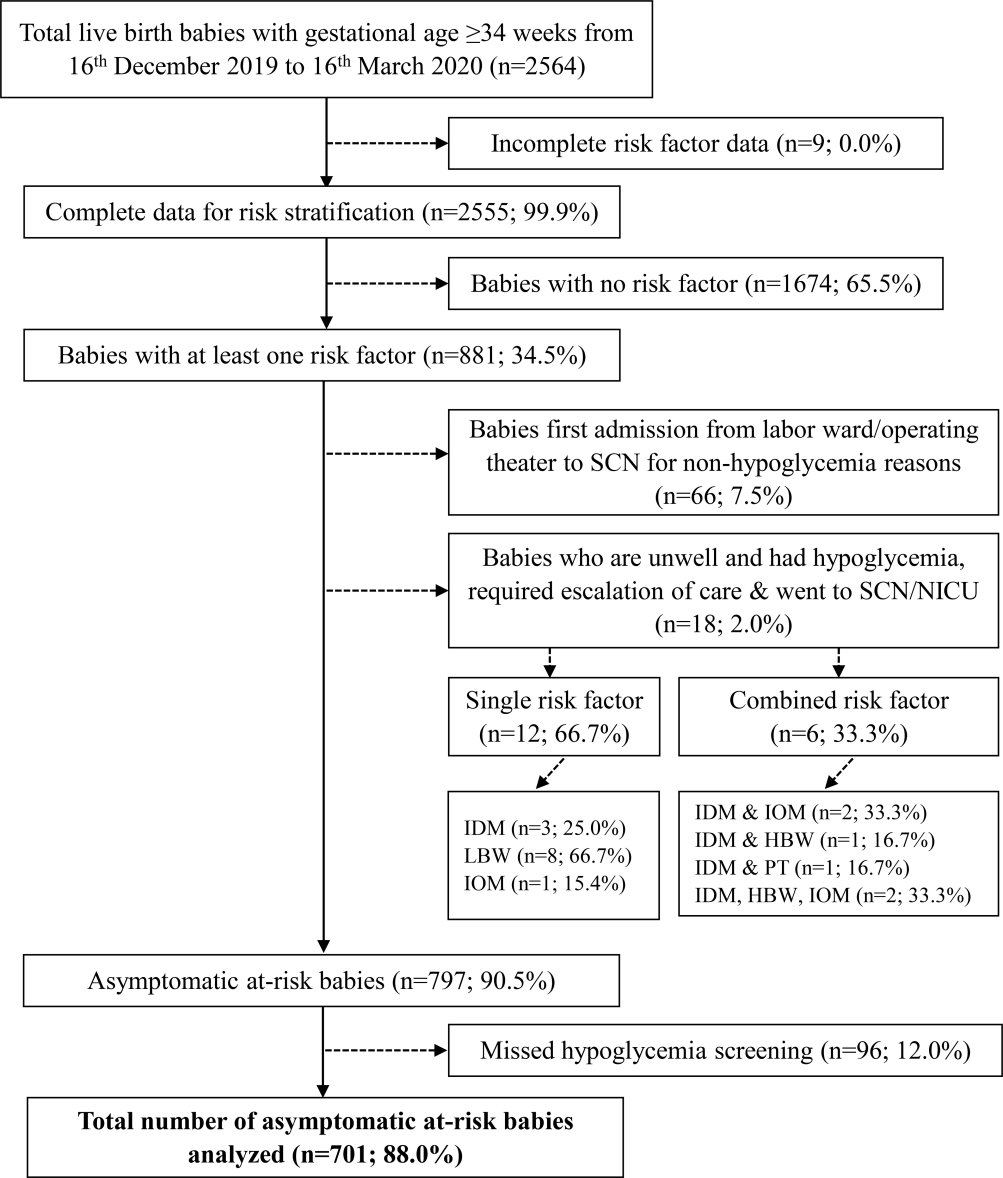
Flow diagram of study population and selection. Asymptomatic infants refer to those who did not have any clinical signs suggestive of hypoglycemia. SCN, special care nursery; NICU, neonatal intensive care unit; IDM, infant of mother with gestational diabetes mellitus/pre-existing diabetes; LBW, term infants with low birth weight (<2600 g); IOM, infant of mother with obesity; HBW, term infants with high birthweight (>4000g); PT, preterm birth (defined as infants born at gestational age < 37 weeks).

During the study period, 2,564 infants were born. After excluding those who (i) had incomplete risk factor data, (ii) had no risk factors, (iii) required urgent escalation of care to Level 2 or Level 3 neonatal care because they were unwell at birth with or without hypoglycemia, and (iv) had missing blood glucose profile data, a study population of 701 infants was derived (**Figure 1**). All 701 infants had at least one glucose measurement within the first 24 hours of life. Among them, 670 infants (95.6%) completed all glucose readings throughout the 24-hour observation period. **Table 1** summarizes the maternal and infant characteristics across the different risk factor groups. At 2 hours post-birth, majority of the infants (n=432, 61.6%) received only breastmilk. By 24 hours, majority (n=440, 63.3%) had received mixed-feeding (both breast milk and formula), while the rest were exclusively breast-fed (n=94, 13.5%) or formula-fed (n=161, 23.2%).

**Table 1:** Maternal and infant baseline characteristics stratified by birth size, maternal metabolic status, and gestational age.

Characteristic	All (n=701)	Birth size				IDM			IOM			Gestational age at birth		
		SGA (n=154; 22.0%)	AGA (n=474; 67.6%)	LGA (n=73; 10.4%)	*p*	No (n=350; 49.9%)	Yes (n=351; 50.1%)	*p*	No (n=537; 76.6%)	Yes (n=164; 23.4%)	*p*	Term (n=584; 83.3%)	Pre-term (n=117; 16.7%)	*p*
Maternal age, years	31.68 ± 5.28	29.71 ± 5.75	32.28 ± 5.04	31.90 ± 4.79	<0.001	30.26 ± 5.42	33.09 ± 4.74	<0.001	31.62 ± 5.43	31.87 ± 4.79	0.579	31.67 ± 5.19	31.72 ± 5.75	0.934
Maternal ethnicity														
Chinese	254 (36.2)	59 (38.3)	181 (38.2)	14 (19.2)	0.026	126 (36.0)	128 (36.5)	0.118	228 (42.5)	26 (15.9)	<0.001	204 (34.9)	50 (42.7)	0.441
Malay	208 (29.7)	47 (30.5)	137 (28.9)	24 (32.9)		117 (33.4)	91 (25.9)		132 (24.6)	76 (46.3)		178 (30.5)	30 (25.6)	
Indian	87 (12.4)	18 (11.7)	60 (12.7)	9 (12.3)		39 (11.1)	48 (13.7)		61 (11.4)	26 (15.9)		73 (12.5)	14 (12.0)	
Others	152 (21.7)	30 (19.5)	96 (20.3)	26 (35.6)		68 (19.4)	84 (23.9)		116 (21.6)	36 (22.0)		129 (22.1)	23 (19.7)	
Maternal first-trimester BMI, kg/m^2^	27.10 ± 6.67	24.06 ± 6.24	27.52 ± 6.63	30.58 ± 5.33	<0.001	26.75 ± 7.28	27.44 ± 6.00	0.178	24.19 ± 4.23	36.42 ± 3.95	<0.001	27.48 ± 6.67	25.14 ± 6.36	<0.001
Maternal GDM (Yes)	298 (44.2)	22 (15.1)	248 (54.0)	28 (40.6)	<0.001	0 (0.0)	298 (86.6)	<0.001	248 (48.2)	50 (31.4)	<0.001	277 (49.1)	21 (19.1)	<0.001
Maternal pre-existing DM (Yes)	25 (3.6)	2 (1.3)	15 (3.2)	8 (11.3)	0.003	0 (0.0)	25 (7.2)	<0.001	17 (3.2)	8 (4.9)	0.439	22 (3.8)	3 (2.7)	0.783
Birth weight, kg	2.94 ± 0.55	2.36 ± 0.19	2.97 ± 0.39	3.93 ± 0.33	<0.001	2.75 ± 0.57	3.12 ± 0.45	<0.001	2.85 ± 0.53	3.21 ± 0.50	<0.001	3.01 ± 0.54	2.57 ± 0.42	<0.001
Gestational age at birth, weeks	38.12 ± 1.26	38.11 ± 1.06	38.07 ± 1.31	38.43 ± 1.34	0.103	37.85 ± 1.34	38.39 ± 1.12	<0.001	37.98 ± 1.28	38.56 ± 1.11	<0.001	38.51 ± 0.96	36.15 ± 0.55	<0.001
Baby’s sex (Male)	347 (49.5)	58 (37.7)	254 (53.6)	35 (47.9)	0.003	167 (47.7)	180 (51.3)	0.385	265 (49.3)	82 (50.0)	0.955	274 (46.9)	73 (62.4)	0.003
Type of first feed at 2hrs of life														
BF	432 (61.6)	93 (60.4)	297 (62.7)	42 (57.5)	0.600	206 (58.9)	226 (64.4)	0.311	337 (62.8)	95 (57.9)	0.527	375 (64.2)	57 (48.7)	0.007
FF	244 (34.8)	58 (37.7)	158 (33.3)	28 (38.4)		130 (37.1)	114 (32.5)		181 (33.7)	63 (38.4)		190 (32.5)	54 (46.2)	
Mixed	25 (3.6)	3 (1.9)	19 (4.0)	3 (4.1)		14 (4.0)	11 (3.1)		19 (3.5)	6 (3.7)		19 (3.3)	6 (5.1)	
Feeding status over 24hrs of life														
Fully BF	94 (13.5)	17 (11.3)	70 (14.8)	7 (9.7)	0.076	47 (13.6)	47 (13.5)	0.664	74 (13.9)	20 (12.4)	0.179	83 (14.3)	11 (9.6)	0.015
Fully FF	161 (23.2)	47 (31.1)	99 (21.0)	15 (20.8)		85 (24.6)	76 (21.8)		115 (21.5)	46 (28.6)		123 (21.2)	38 (33.3)	
Mixed	440 (63.3)	87 (57.6)	303 (64.2)	50 (69.4)		214 (61.8)	226 (64.8)		345 (64.6)	95 (59.0)		375 (64.5)	65 (57.0)	
Number of glucogel doses required														
0	612 (87.3)	129 (83.8)	418 (88.2)	65 (89.0)	0.085	305 (87.1)	307 (87.5)	0.898	466 (86.8)	146 (89.0)	0.450	511 (87.5)	101 (86.3)	0.728
1	78 (11.1)	24 (15.6)	49 (10.3)	5 (6.8)		39 (11.1)	39 (11.1)		66 (12.3)	12 (7.3)		64 (11.0)	14 (12.0)	
2	11 (1.6)	1 (0.6)	7 (1.5)	3 (4.1)		6 (1.7)	5 (1.4)		5 (0.9)	6 (3.7)		9 (1.5)	2 (1.7)	

Values are in means ± SDs for continuous variables or n (%) for categorical variables. SGA, small for gestational age, AGA, appropriate for gestational age, LGA, large for gestational age; IDM, infants of diabetic mothers; IOM, infants of obese mothers; BMI, body mass index; GDM, gestational diabetes mellitus; DM, diabetes mellitus; BF, breast-fed; FF, formula-fed; Mixed, breast-fed and formula-fed. Preterm infants are defined as those born at gestational age of less than 37 weeks.

χ^2^ or Fisher exact tests were used for categorical variables and one-way analysis of variance was used for continuous variables to compare 3 groups and independent t-test for 2 groups.

### Risk factor distribution

**Figure 2:**
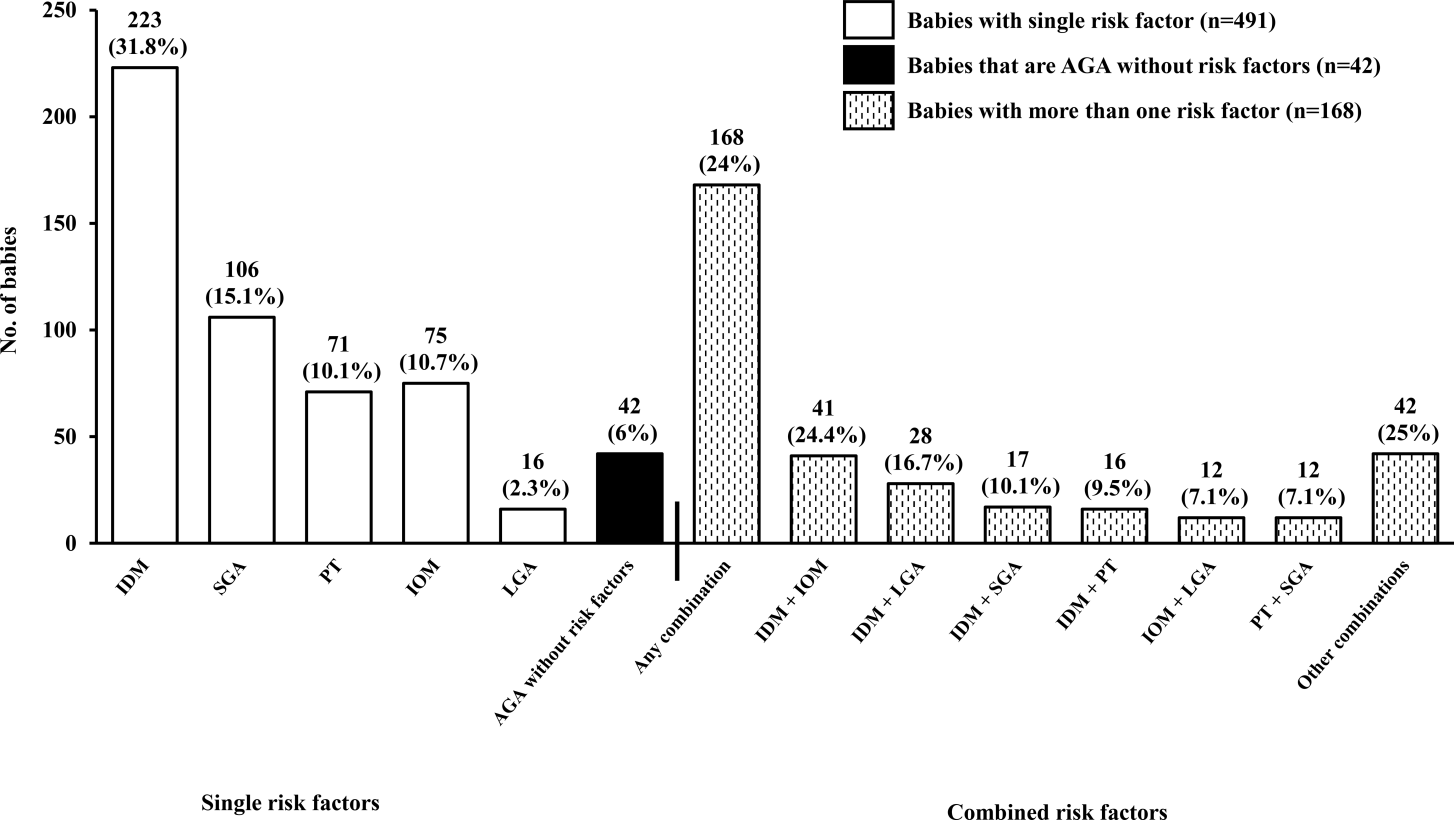
Distribution of risk factors among participants. Open bars represent infants with a single risk factor (n=491, 70.0%), black bars denote AGA infants without risk factors (n=42, 6.0%), and hatched bars represent infants with multiple risk factors (n=168, 24.0%). Percentages for single risk factors are calculated relative to the total study cohort (n=701), whereas percentages for multiple risk factor subgroups are calculated within the 168 infants with combined risk factors. AGA, appropriate for gestational age; LGA, large for gestational age; SGA, small for gestational age; IDM, infant of diabetic mother (GDM/pre-existing diabetes); IOM, infant of mother with obesity; PT, preterm birth (defined as infants born at gestational age < 37 weeks).

**Figure 2** illustrates the distribution of risk factors among the cohort, distinguishing between infants with single and combined risk factors. The majority (70.0%) had only one identifiable risk factor, whereas 24.0% had more than one. Among the infants with single risk factors, the most commonly represented independent single risk factors were: IDM (31.8%), followed by SGA (15.1%), IOM (10.7%), PT (10.1%), and LGA (2.3%). Although the protocol was designed to screen only at-risk infants, 6.0% of the cohort were reclassified as AGA based on IG21 growth standards. These infants were initially enrolled due to birth weights below 2600g or above 4000g, but had no other risk factors and were subsequently determined to be within the normal birth weight range for gestational age by IG21 growth standards. The commonest risk factor combination was IDM and IOM (24.4%).

### Glucose trends in at-risk infants

**Table 2** summarizes the incidence of hypoglycemia across risk factor groups from 2 to 24 hours after birth. While numerical differences in hypoglycemia rates were observed across groups at the <3.0 and <2.6 mmol/L thresholds, these were not statistically significant. A statistically significant difference between groups was observed only at the <2.2 mmol/L threshold (p = 0.024). At this threshold, the highest incidences were seen in preterm infants (5.6%), those with combined risk factors (5.4%), and SGA infants (4.7%). Together, these three groups accounted for 18 of the 21 hypoglycemia cases at this threshold.

**Table 2:** Incidence of hypoglycemia in at-risk babies stratified by risk factors according to hypoglycemia thresholds of < 3.0 mmol/L, < 2.6 mmol/L and < 2.2mmol/L.

Hypoglycemia episodes	All (n=701)	Single-risk factors (n=491; 70.0%)					AGA (n=42; 6.0%)	Combined risk-factors (n=168; 24.0%)	p-value
		IDM (n=223; 31.8%)	LGA (n=16; 2.3%)	SGA (n=106; 15.1%)	Pre-term (n=71; 10.1%)	IOM (n=75; 10.7%)			
< 3.0 mmol/L at least once in 24 hrs	114 (16.3)	29 (13.0)	1 (6.2)	24 (22.6)	12 (16.9)	12 (16.0)	5 (11.9)	31 (18.5)	0.295
< 2.6 mmol/L at least once in 24 hrs	51 (7.3)	10 (4.5)	0	10 (9.4)	5 (7.0)	7 (9.3)	3 (7.1)	16 (9.5)	0.391
< 2.2 mmol/L at least once in 24 hrs	21 (3.0)	1 (0.4)	0	5 (4.7)	4 (5.6)	1 (1.3)	1 (2.4)	9 (5.4)	0.024
@ 2 hrs									
< 3.0 mmol/L	57 (8.1)	17 (7.6)	0	11 (10.4)	3 (4.2)	5 (6.7)	0	21 (12.5)	0.065
< 2.2 mmol/L	15 (2.1)	1 (0.4)	0	4 (3.8)	2 (2.8)	1 (1.3)	0	7 (4.2)	0.123
@ 6 hrs									
< 3.0 mmol/L	27 (3.9)	7 (3.2)	0	6 (5.8)	3 (4.2)	3 (4.1)	2 (4.8)	6 (3.7)	0.919
< 2.6 mmol/L	12 (1.7)	1 (0.5)	0	4 (3.9)	1 (1.4)	1 (1.4)	1 (2.4)	4 (2.5)	0.294
@ 12 hrs									
< 3.0 mmol/L	17 (2.4)	3 (1.4)	0	1 (1.0)	3 (4.2)	3 (4.1)	1 (2.4)	6 (3.8)	0.457
< 2.6 mmol/L	5 (0.7)	1 (0.5)	0	0	1 (1.4)	2 (2.7)	0	1 (0.6)	0.397
@ 18 hrs									
< 3.0 mmol/L	6 (0.9)	0	1 (6.2)	1 (1.0)	0	1 (1.3)	1 (2.4)	2 (1.2)	0.059
< 2.6 mmol/L	2 (0.3)	0	0	0	0	1 (1.3)	0	1 (0.6)	0.390
@ 24 hrs									
< 3.0 mmol/L	17 (2.4)	3 (1.4)	0	5 (4.7)	3 (4.2)	2 (2.7)	2 (4.8)	2 (1.2)	0.224
< 2.6 mmol/L	4 (0.6)	1 (0.5)	0	0	1 (1.4)	0	2 (4.8)	0	0.047

Values are in n (%). This tables indicates repeated incidences of hypoglycemia. IDM, infants of diabetic mother; SGA, small for gestational age; LGA, large for gestational age; IDM, infants of diabetic mothers; IOM, infants of obese mother; AGA, appropriate for gestational age. Preterm infants are defined as those born at gestational age of less than 37 weeks.

**Figure 3:**
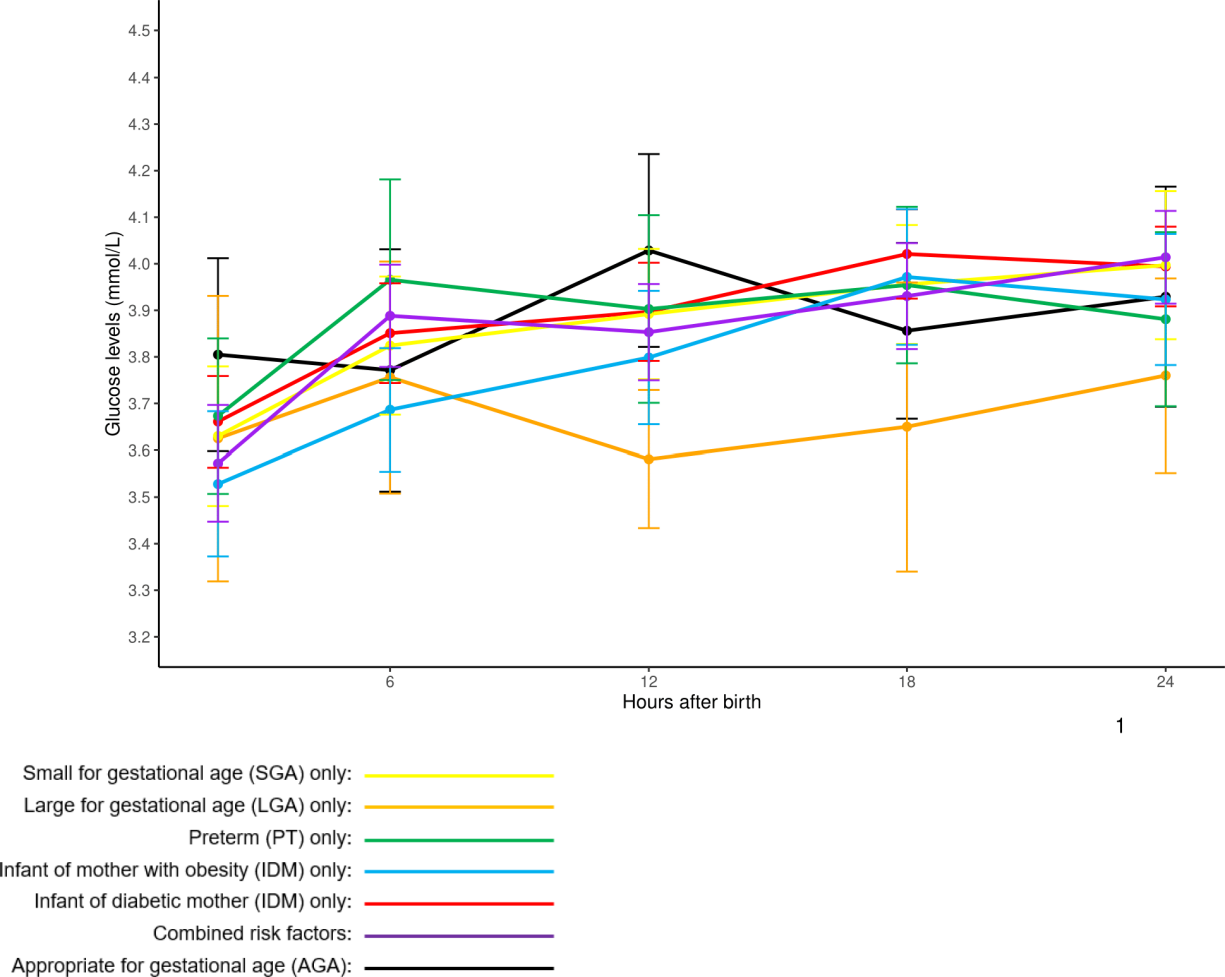
Overall mean glucose profiles of at-risk babies stratified by single and combined risk factors. Glucose readings are reported in mean ± 95% confidence intervals.

Mean glucose levels generally showed an upward trend across all risk groups from 2 to 24 hours post-birth. AGA infants without risk factors had the highest mean glucose levels at 2 hours (3.80 ± 0.69 mmol/L), while infants with combined risk factors had the highest levels at 24 hours (4.01 ± 0.64 mmol/L). On the other hand, IOM infants had the lowest mean glucose levels at 2 hours (3.53 ± 0.69 mmol/L), whereas IDM infants exhibited the highest mean glucose levels at 18 hours (4.02 ± 0.72 mmol/L). Among the relatively small group of LGA infants, only one case of hypoglycemia was observed, and there was a dip in mean glucose levels at 12 hours (3.58 ± 0.30 mmol/L), down from 3.76 ± 0.51 mmol/L at 6 hours. This trend was not observed in other risk factor groups. Hypoglycemia episodes most frequently occurred at 2 hours post-birth across all risk factor groups. Glucose stabilization trends across risk factor groups and feeding types are further illustrated in **Figure 3**, which depicts overall glucose trends, and **Supplementary Figure 1**, which shows trends stratified by feeding type.

### Risk factor associations with hypoglycemia incidence

**Table 3** shows the associations between risk factors and the incidence of hypoglycemia at 3.0 mmol/L, 2.6 mmol/L, and 2.2 mmol/L thresholds. SGA infants had a significantly higher likelihood of experiencing hypoglycemia within the first 24 hours compared to AGA infants, at both the 3.0 mmol/L threshold (OR 1.76; 95% CI 1.10, 2.76; P = 0.016) and the 2.2 mmol/L threshold (OR 2.88; 95% CI 1.12, 7.28; P = 0.024).

**Table 3:** Odds Ratios for Hypoglycemia within 24 hours of birth stratified by risk factors.

Outcome and risk factor	Reference	Events	Odds ratio (95% CI)	*p*
< 3.0 mmol/L		114		
IDM	Non-IDM	54	0.88 (0.59, 1.31)	0.528
LGA	AGA	11	1.06 (0.51, 2.04)	0.870
SGA	AGA	35	1.76 (1.10, 2.76)	0.016
Pre-term	Term	23	1.33 (0.78, 2.17)	0.277
IOM	Non-IOM	23	0.80 (0.48, 1.29)	0.376
Combined risk factors	Single risk factor	31	1.20 (0.75, 1.88)	0.440
< 2.6 mmol/L		51		
IDM	Non-IDM	21	0.68 (0.38, 1.20)	0.189
LGA	AGA	6	1.37 (0.50, 3.22)	0.496
SGA	AGA	16	1.78 (0.92, 3.33)	0.077
Pre-term	Term	11	1.41 (0.67, 2.75)	0.334
IOM	Non-IOM	11	0.89 (0.43, 1.73)	0.749
Combined risk factors	Single risk factor	16	1.51 (0.79, 2.79)	0.198
< 2.2 mmol/L		21		
IDM	Non-IDM	7	0.49 (0.18, 1.19)	0.127
LGA	AGA	2	1.31 (0.20, 5.09)	0.733
SGA	AGA	9	2.88 (1.12, 7.28)	0.024
Pre-term	Term	9	3.97 (1.59, 9.62)	0.002
IOM	Non-IOM	3	0.54 (0.12, 1.61)	0.324
Combined risk factors	Single risk factor	9	2.47 (0.98, 6.08)	0.049

SGA, small for gestational age; LGA, large for gestational age; AGA, appropriate-or-gestational age; CI; confidence intervals; IDM, infants of diabetic mothers; IOM, infants of obese mother.

Preterm birth was also significantly associated with an increased risk of hypoglycaemia at the 2.2 mmol/L threshold (OR 3.97; 95% CI 1.59, 9.62; P = 0.002). Additionally, infants with more than one risk factor were 2.5 times more likely to experience hypoglycemia (OR 2.47; 95% CI 0.98, 6.08; P = 0.049) compared to those with only one risk factor at the 2.2 mmol/L threshold.

## Discussion

This prospective study of 701 at-risk infants, who were predominantly mixed or fully formula-fed by 24 hours of life, demonstrates that a large majority (83.7%) had BG ≥3.0mmol/L in the first 24 hours of life when they were systematically screened. This means that the incidence of hypoglycemia using the operational threshold of BG <3.0mmol/L in our study was 16.3%, or approximately 1 in 7 at-risk babies. Consistent with other studies (23), the highest hypoglycemia incidence (22.6%) involved single risk factor SGA infants, which aligns with their limited glycogen reserves and reduced gluconeogenic ability; however, only 9.4% had BG <2.6mmol/L. In contrast, the low hypoglycemia incidence in single risk factor LGA babies (6.2%) is comparatively unexpected (24) – only one case of hypoglycaemia was recorded among 16 babies, which may either reflect a sample size limitation or that routine screening in this subgroup may require further evaluation. Unsurprisingly, single risk factor preterm infants demonstrated greater risk of developing more severe hypoglycemia <2.2mmol/L compared to term infants, with an odds ratio of 3.97, emphasizing the impact of prematurity on metabolic adaptation (25). Altogether, these findings suggest the need to refine clinical protocols to tailor screening and strategies to distinct risk profiles of at-risk infants, ensuring early identification while minimizing unnecessary interventions.

While most studies on neonatal hypoglycemia focus on individual risk factors such as SGA, preterm birth, and IDM (25, 26), limited research has examined the compound effects of combined risk factors on hypoglycemia incidence. Our study demonstrated that infants with more than one risk factor had a higher incidence of hypoglycemia (31/168, 18.5% at <3.0 mmol/L) compared to those with a single risk factor (78/491, 15.9% at <3.0mmol/L), although this was not statistically significant. This aligns with findings suggesting that overlapping metabolic vulnerabilities, such as impaired glycogen storage and altered insulin regulation, exacerbate hypoglycemia risk (3, 7). Notably, our study included single risk factor infants of mothers with obesity, a group not traditionally classified as at risk for hypoglycemia, yet we observed hypoglycemia rates comparable to those of infants of diabetic mothers. This finding underscores the potential impact of intrauterine metabolic programming on neonatal glucose regulation (9, 13). The systematic classification of risk factors in our cohort highlights the need for a more nuanced approach to neonatal glucose monitoring, moving beyond a uniform screening protocol to a risk-adapted strategy that accounts for overlapping metabolic vulnerabilities. Further research should explore how different combinations of risk factors influence long-term metabolic and neurodevelopmental outcomes (2, 4).

Among studies examining the incidence of hypoglycemia in at-risk newborns (5, 8, 10, 15, 27, 28), the study by Harris et al. in 2012 stands out for its prospective design, large sample size and rigorous glucose measurement methodology. Our study reports substantially lower hypoglycemia rates (16.5% with BG <3.0mmol/L; 7.3% with BG <2.6mmol/L) compared to the study by Harris et al. (10), where 50.5% of 514 infants had at least one BG <2.6mmol/L. This is reflective of methodological differences. In the study by Harris et al., glucose testing began at 1 hour of life for all participants, within the time frame of the expected glucose nadir, potentially raising hypoglycemia rates, while our participants started at 2 hours, reducing the measurement of early transient lows. Harris et al. employed the glucose oxidase method and more flexible glucose testing (median 9 tests over 48 hours, range 1 to 21), while we applied a fixed 5-test schedule using a point-of-care glucose dehydrogenase method. Despite point-of-care testing’s potential of overestimating BG by up to 15% (29), we still found fewer hypoglycemia cases. Harris et al. found similar hypoglycemia rates across at-risk groups, while we found variability, including a 16% incidence in infants of mothers with BMI ≥33kg/m^2^. Our structured approach and the use of updated growth standards highlight how the timing of BG tests and methods can influence hypoglycemia detection. While Harris et al. suggested simplifying screening, our study complements that by highlighting the potential value of a risk-classified approach, where screening intensity is guided by the type and combination of clinical risk factors.

One unique feature of our study is the inclusion of infants of obese mothers, who are not customarily considered at-risk of hypoglycemia, allowing comparisons between single risk factor IDM, LGA and IOM. We observed that the hypoglycemia incidence was similar in IOM and IDM. Excess maternal weight gain because of increased caloric intake, even when glucose tolerance is normal (14), provides a plausible biological mechanism of this condition (30). Maternal obesity has independently been associated with an increased risk of gestational diabetes, largely mediated by underlying insulin resistance and metabolic dysfunction (31). In Singapore, all pregnant women undergo routine gestational diabetes screening with an OGTT at 24–28 weeks. Nonetheless, as with any screening strategy, some cases of maternal hyperglycemia may be missed. As maternal glucose data were not collected in this study, future research should incorporate metabolic markers to better understand the association between maternal obesity and neonatal hypoglycemia. Unlike IOM, LGA babies showed remarkably low incidence of hypoglycemia, which support prior findings that routine screening may not be necessary in this group, unless combined with other risk factors such as gestational diabetes (27, 32). However, given the relatively small sample size of LGA infants (n=73), and an even smaller number of single risk factor LGA (n=16), it remains uncertain whether the low hypoglycemia incidence in single risk factor LGA infants reflects a true absence of increased risk. These “LGA only” infants comprised only 22% (16 of 73) of all infants born LGA. Hence, larger cohorts will need to be studied to clarify whether “LGA only” infants—particularly those without maternal risk factors or relevant family history—are at inherently lower risk of hypoglycemia, or whether certain subsets within this group still carry metabolic vulnerabilities (33).

Future research should prioritize establishing evidence-based glucose thresholds for different at-risk populations, including single risk factor SGA, preterm, LGA, IDM, and IOM infants, as current guidelines lack universal consensus (7, 34). Additionally, understanding the long-term neurodevelopmental impacts of neonatal hypoglycemia remains critical, as specific glycemic patterns have been linked to adverse outcomes such as cognitive deficits and motor skill delays (25). While emerging technologies like continuous glucose monitoring (CGM) hold potential for advancing neonatal care, further studies are needed to validate their clinical effectiveness and assess their feasibility in real-world settings (35–37). Ultimately, refining hypoglycemia definitions and exploring individualized management strategies will be essential for improving both short and long-term outcomes for at-risk infants (38).

Our study has several notable strengths. Firstly, the prospective design and comprehensive glucose monitoring protocol allowed for a robust assessment of glucose patterns in a large cohort of at-risk infants. Additionally, our study stratified infants based on single and combined risk factors, providing nuanced insights into the differential risk profiles of single risk factor SGA, LGA, preterm, IDM, and IOM infants. The use of multiple glucose thresholds (<3.0, <2.6, and <2.2 mmol/L) adds depth to our findings, offering further perspectives on the spectrum of hypoglycemia incidence and severity across risk factor groups.

However, we do recognize the limitations of our study. The single-centre design may limit the generalizability of our findings to other settings with different clinical practices, population characteristics, and feeding protocols. Furthermore, while our cohort included a substantial number of at-risk infants, the relatively small sizes of some risk groups, such as LGA and combined risk factor groups like IDM/IOM and IDM/SGA, constrained further analyses and limited the statistical power to explore interactions between multiple risk factors. Although our AGA reference group was defined according to INTERGROWTH-21st standards (10th–90th percentile), it included infants with birth weight >4 kg and <2.6 kg; thus, generalizability and applicability of our findings should be interpreted with caution. Our findings should also be interpreted in the context of feeding practices within our study cohort. With a sizeable proportion of newborns receiving mixed or formula feeding by 24 hours, our population may not fully reflect the metabolic responses of exclusively breastfed neonates. Early formula supplementation has been associated with altered glucose homeostasis and reduced exclusive breastfeeding rates at discharge (39, 40). Future studies should explore how different feeding practices influence neonatal glucose stabilization and hypoglycemia risk. Lastly, the absence of long-term follow-up data in our cohort precludes a direct examination of the neurodevelopmental outcomes associated with hypoglycaemic events across different risk factor groups. Given the potential impact of early-life hypoglycemia on long-term cognitive and motor development, future studies should aim to include longitudinal follow-up to better understand these associations.

In conclusion, our study highlights distinctive glucose stabilization patterns and varying risks of hypoglycemia in at-risk infants, emphasizing the need for tailored screening and management protocols. The findings underscore the importance of adapting clinical practices based on single and combined risk factors. Future research should prioritize establishing standardized glucose thresholds to define neonatal hypoglycaemia and investigating the long-term neurodevelopmental impacts of neonatal hypoglycemia in high-risk infants to optimize care.

## Data Availability

The original contributions presented in the study are included in the article/**Supplementary Material**. Further inquiries can be directed to the corresponding authors.

## Supplementary material

Supplementary Figure 1 Overall mean glucose profiles of at-risk babies stratified by feeding type throughout 24 hours of life. Results are reported in mean ± 95% confidence intervals. BF, breastfed; FF, formula-fed.
