# Supplementary material for: Glucose Levels in At-risk Newborns (GLEAN): a prospective cohort study on glucose profiles in infants at risk of hypoglycemia
Source: Front Endocrinol (Lausanne). 2025 Aug 8;16:1599366. doi: 10.3389/fendo.2025.1599366 (PMC12370509; doi:10.3389/fendo.2025.1599366)
Supplement: Supplementary Figure 1 — Overall mean glucose profiles of at-risk babies stratified by feeding type throughout 24 hours of life. Results are reported in mean ± 95% confidence intervals. BF, breastfed; FF, formula-fed. [file DataSheet1.docx]

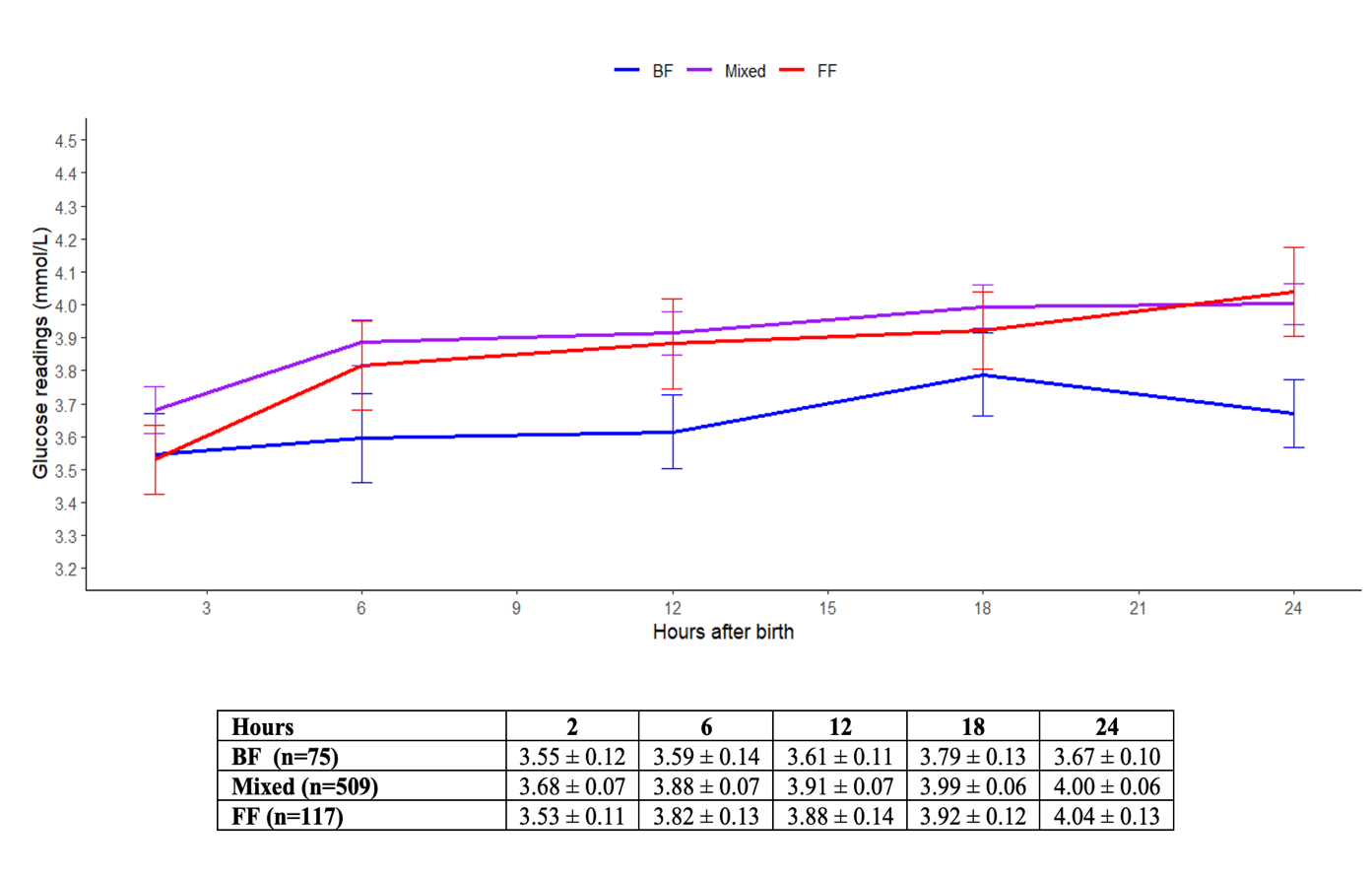


Supplementary Figure 1. Overall mean glucose profiles of at-risk babies stratified by feeding type throughout 24 hours of life.

Results are reported in mean ± 95% confidence intervals. Abbreviations: BF, breastfed; FF, formula-fed.
